# Evaluation of probe-based ultra-sensitive detection of miRNA using a single-molecule fluorescence imaging method: miR-126 used as the model

**DOI:** 10.3389/fbioe.2023.1081488

**Published:** 2023-01-24

**Authors:** Longkai Liu, Xiaoning Wang, Yan Li, Jianwei Liu

**Affiliations:** ^1^ School of Pharmacy, Department of Chemistry, Institutes of Biomedical Sciences, Fudan University, Shanghai, China; ^2^ Department of Pharmacy, Qingdao Municipal Hospital, Qingdao, China

**Keywords:** single molecule imaging, fluorescence, microRNA, DNA probe, cancer biomarker detection

## Abstract

This study proposed a new detection method of miRNA based on single-molecule fluorescence imaging, a method that has been successfully developed to measure the light signal of individual molecules labeled with proper fluorophores. We designed probes 1 and 2 to be labeled with Cy5 dye and BHQ2 quencher at the 3′terminals, respectively. Probe 1 consisted of two parts, the longer part complementary to miR-126 and the shorter part complementary to probe 2. After hybridization, miR-126 bound to probe 1 by replacing probe 2 and assembled into a double-stranded DNA with probe 1. The abundance of miR-126 was quantified by detecting image spots of Cy5 dye molecules from probe 1/miR-126 complexes. MiR-126 single-molecule imaging method showed high specificity and sensitivity for miR-126 with a detection limit of 50 fM. This method has good selectivity for miR-126 detection with 2.1-fold, 8.8-fold, and 26.9–41.3-fold higher than those of single-base mismatched miR-126, three-base mismatched miR-126 and non-complementary miRNAs (miR-221, miR-16, miR-143 and miR-141). The method to detect miR-126 was validated in breast cancer cell lines. Our single-molecule miRNA imaging showed high specificity and sensitivity for miRNAs. By changing the base pair sequence of the designed probes, our method would be able to detect different miRNAs.

## Highlights

1) miR-126 assembled into a double-stranded DNA with probe 1 *via* replacing probe 2.
2) Two probes not only captured miRNA molecules but also eliminate false positive signals.
3) Single-molecule fluorescence imaging showed a detection limit of 50 fM for miR-126.
4) The miR-126 of breast cancer cell vs. control cell was detected by this method.

## Introduction

MicroRNAs (miRNAs) are highly conserved non-coding short RNA molecules with a number of 19–25 nucleotides, which cause the post-transcriptional silencing of target genes. Numerous investigations have proved that miRNAs play a crucial part in the regulation of various cellular processes such as metabolism apoptosis, proliferation, and development. Therefore, miRNAs are strictly regulated in the organism and play a role in many diseases (Lu and Rothenberg, 2013; Zhang et al., 2014; Dissanayake and Inoue, 2016; Liu et al., 2016). For example, miR-126 inhibits the progression of certain tumors by inhibiting the translation of related target genes (Chakraborty et al., 2016) and then suppressing the proliferation, migration and invasion of tumors, which are very important in tumor biology. Specifically, tumorigenesis and progression of breast cancer was manipulated by miRNAs among which miR-126 exhibited an inhibiting action on the metastasis of human breast cancer cell MDA-MB-231 in severe combined immunodeficiency mice threough simultaneous targeted silencing of angiogenesis- and metastasis-associated genes (Volinia et al., 2006; Ma et al., 2007; Tavazoie et al., 2008; Png et al., 2012). However, miRNAs face the challenge of detection and analysis due to the limitation of some of their inherent properties, such as high sequence homology, low abundance, instability and short sequence length.

Traditional methods in detecting miRNAs include Northern blotting (Kim et al., 2010), solid phase hybridization, RT-PCR (Feng et al., 2009) and microarray (Thomson et al., 2004). However, some negative issues (such as complicated procedures and high costs) of these methods, especially low sensitivity, limit their application. To address these problems, some new methods are being developed, such as isothermal amplification, electrochemical methods and nanoparticle probes (Valoczi et al., 2004; Chen et al., 2005; Li and Ruan, 2009; Hwang et al., 2010; Koscianska et al., 2011; Li et al., 2011; Zhang and Zeng, 2011; Li et al., 2014). The common strategy of the newly developed methods is to amplify the signal multiple times and then use certain sensitive signal-detecting units to achieve good detection sensitivity. However, the signal amplification strategy raises the complexity of the analysis procedure while the non-uniformity of signal amplification introduces extra error. Therefore, there is a great need to develop the simple and highly sensitive detection method of miRNAs.

In the past few decades, the development of optics, detectors and fluorophores has caused huge progress in single-molecule optical imaging methods that dramatically improved the detection limit of biomolecular measurement even without the need for enzymatic reactions and signal amplification (Liu et al., 2013; Farka et al., 2017; Smith et al., 2018a). Compared with traditional methods, the single-molecule detection method not only improves the detection limit and sensitivity but also enables the measurement of molecule heterogeneity. By directly observing the dynamics of single molecules in physical or chemical processing, we can understand the change processes and intermediate states of individual molecules (Walt, 2013). Moreover, the single molecule detection method has the advantage of low sample consumption and rapid analysis, making it an ideal platform for biomolecule quantification. Accumulating works have attempted to verify the feasibility of the miRNA single-molecule imaging method, especially with fluorescence imaging (Li et al., 2020; Li et al., 2021). However, the technology has yet to be improved to provide highly specific and sensitive miRNA single-molecule detection methods.

In this work, we demonstrate the capability of the proposed single-molecule fluorescence imaging method for the detection of miRNAs with miR-126 as the model.

## Material and methods

### Reagents

Streptavidin (SA) and PBS buffer without RNase were provided by Sangon Biotech (Shanghai, China). Poly (L-lysine)-poly (ethyleneglycol)-biotin (PLL-PEG-Biotin) and poly (L-lysine)-poly (ethyleneglycol) were provided by Susos AG (Switzerland). The miRNAs and cDNA labeled with fluorescent dyes were synthesized by Sangon Biotech (Shanghai, China) and their sequences were shown in Supplementary Table S1. The growth factors for cell culture were purchased from Sigma (United States). Mediums, horse serum, fetal bovine serum (FBS), and penicillin-streptomycin were provided by Gibco (United States).

### Cell culturing and miRNA extraction

Two human breast cancer cell lines MB-231 and MCF10A were obtained from the Cell Bank of the Chinese Academy of Sciences (Shanghai, China). MCF10A cells were cultured in DMEM/F-12 supplemented with 100 ng/mL cholera toxin, 20 ng/mL EGF, 10 μg/mL insulin, 50 μg/mL hydrocortisone, 5% horse serum, and 1% penicillin-streptomycin. MB-231 cells were cultured in DMEM mixed with 10% FBS and 1% penicillin-streptomycin. Cells were all cultured at 37°C and under a 5% CO_2_ atmosphere. miRNAs extraction was performed using a miRNA extraction kit (Sagon, Shanghai, China) following protocols recommended by the manufacturer. miRNAs were dissolved in DEPC water Sangon Biotech (Shanghai, China) before fluorescent imaging detection.

### Treatment of coverslip and sample cell

Coverslips (Fisher Inc. United States) were prepared by three steps before the experiments as following procedures: first, grease on the coverslip surface was washed off by 1M of potassium hydroxide (KOH) solution by sultrasonication; second, the coverslip was immersed in ethanol and ultrasonicated for 10 min for three times follows by ultrapure water treatment for three times; third, the coverslip was dried by N2 stream.

The sample cell consisted of a combination of a coverslip and a thick slide. The thick slide had a hole with a diameter of 5 mm in the middle and was covered at the bottom by a coverslip through vacuum grease to form a flow cell. The flow sample cell was etched in vacuum plasma (Harrick Plasma. United States) for 10 min. The coverslip was passivated with a mixture of PLL-PEG and PLL-PEG-Biotin (radio of 10:1) for 1 h. After washing three times with PBS, the sample cell was then added with 50 μl SA (0.2 mg/mL) and maintained at room temperature for 15 min. The treated sample cell was kept in a PBS buffer.

### miRNAs labeled by probes

The treated sample cell was added with 50 μl of probe 1 solution (100 pM) and probe 2 solution (10 nM), respectively. Unbounded probe 1 or probe 2 was washed off by PBS buffer. A volume of 50 μl miR-126 solution was added into the sample cell and reacted with probe1/2 mixture for 60 min at 37°C. The sample cell to be detected by single-molecule fluorescence imaging was maintained in 50 μl of PBS buffer. To evaluate the selectivity of the miR-126 single-molecule imaging method, miR-126 in the above detection system was replaced with miR-126 with single gene mismatch, or miR-126 with a three-base mismatch, or non-complementary miRNAs-miR-221, miRNA-16, miRNA-143, and miR-141.

### Single-molecule fluorescent imaging and data processing

The single-molecule miRNA fluorescent imaging was performed in the self-built experiment system referring to a wild-field fluorescence device. The fluorescence of Cy5 dyes was excited at 632 nm by EMCCD (Ixon DU897, Andor Technology, U.K.). To obtain the single-molecule miRNA fluorescence images, 10 regions each imaged in 30 consecutive frames were photographed with an 100 ms exposure. The single-molecule imaging data were analyzed by using a homemade MATLAB (Mathworks Inc. MA, United States) program.

## Results

### Principle and feasibility of miR-126 single-molecule imaging method

**SCHEME 1:**
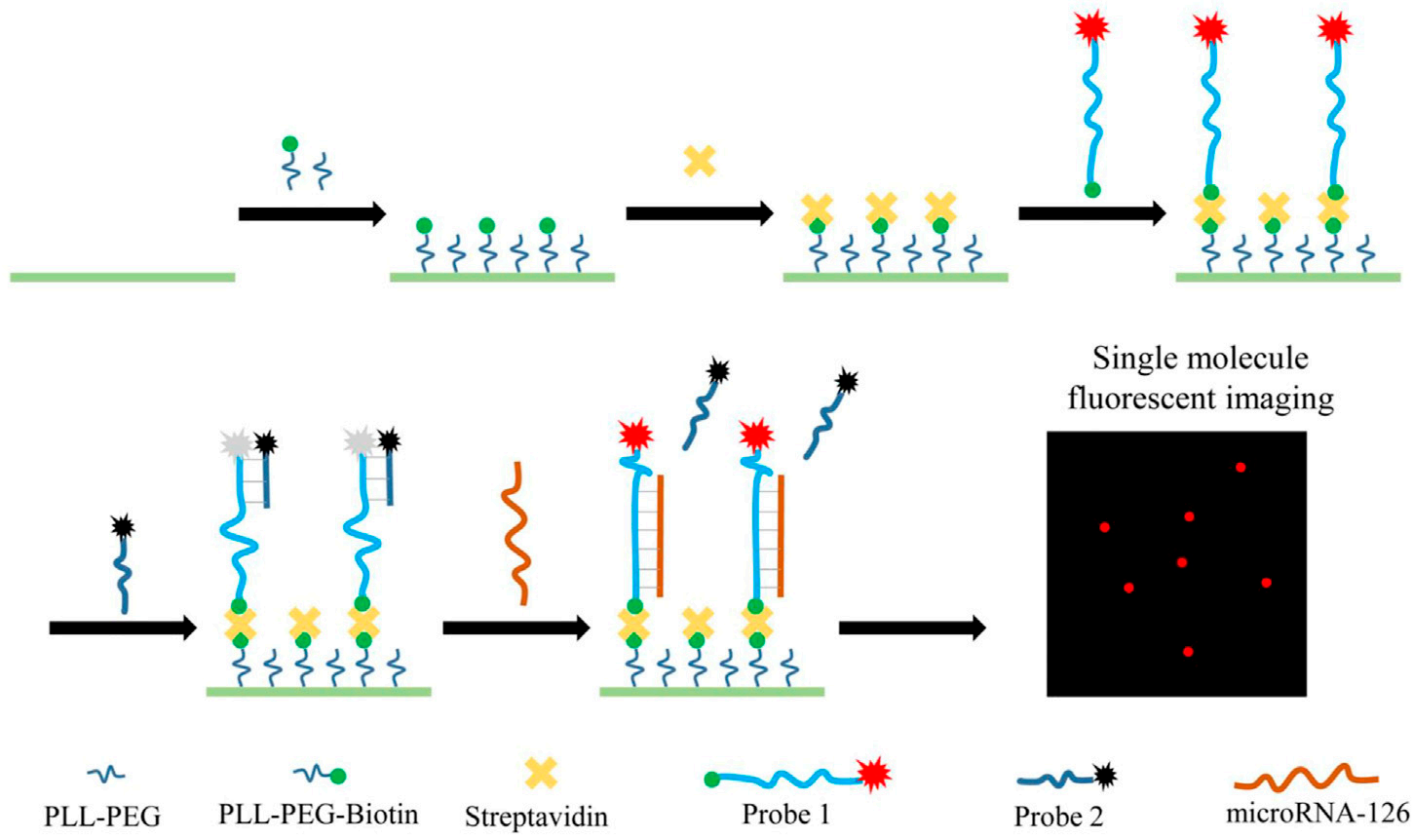
Schematic diagram of single-molecule fluorescence imaging assay.

The principle of this method was shown in Scheme 1. The coverslip was modified by passivation and biotinylation with positive charged PLL−PEG-Biotin and the Cy5-labelled Probe 1 was anchored on the coverslip surface through the specifical binding between SA and biotin. Initially, the quencher-labelled probe 2 bound to probe 1 *via* complementary base pairing and quenched the fluorescence of probe 1. Once the sample having miR-126 was added to the coverslip, the miR-126 molecule would replace the probe 1 bound probe 2 that was designed to have lower affinity to probe 1 than miRNA-126 and assemble a miRNA-126/probe 1 complex. Consequently, the Cy5 dye of probe 1/miR-126 complex recovered from the quenching and generated a fluorescent signal, while the Cy5 dyes of probe 1/probe 2 pairs were quenched. Then the miR-126 abundance was measured by counting Cy5 fluorescent spots on the coverslip surface.

**FIGURE 1:**
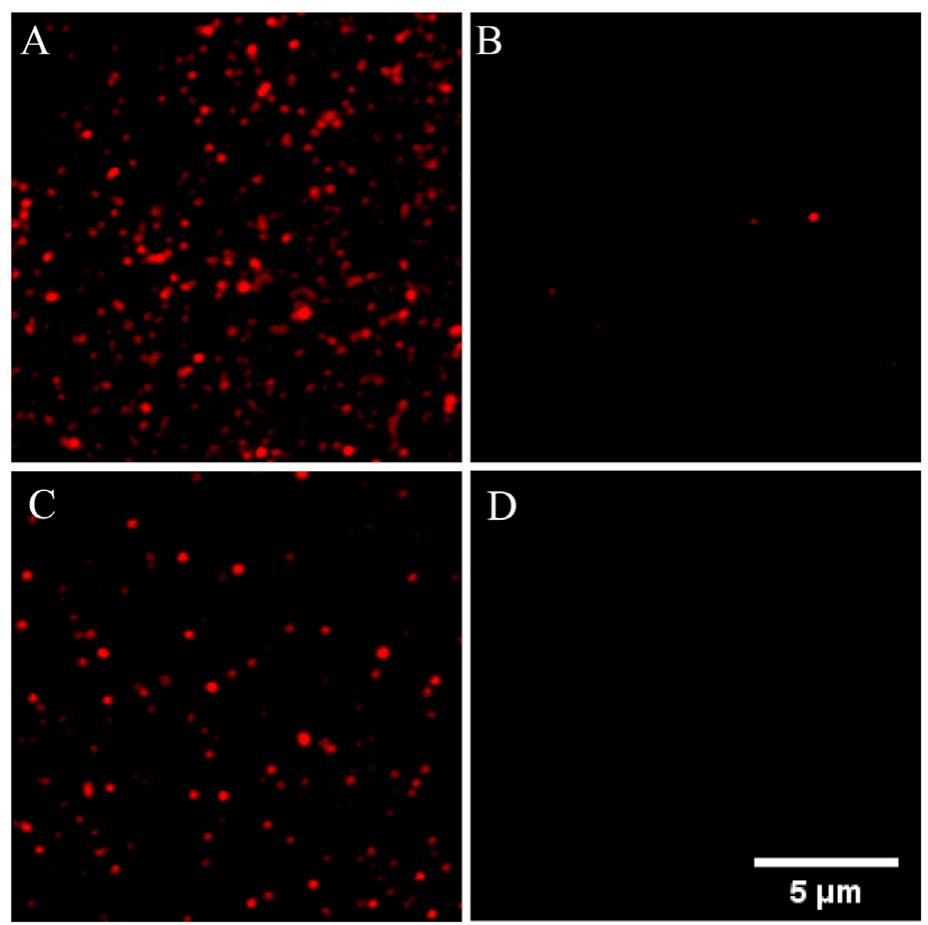
Different single-molecule fluorescence imaging was shown in the PLL-PEG-Biotin-modified sample cells with different treatments. **(A)** The modified sample cells were incubated with SA and probe 1. **(B)** The modified sample cells were incubated with SA and probe 1/2; **(C)** Above sample cell was used for miR-126 detection. **(D)** The modified sample cells were incubated with probe 1. Probe 1: 1 nM; Probe 2: 10 nM; miR-126: 10 pM; Scale bar: 5 μm.

The feasibility of the proposed method in detecting miR-126 was verified in Figure 1. Figure 1A showed a lot of fluorescence spots of Cy5 resulting from the assembly of probe 1 on the coverslip surface. When mixed with probe 2, probe 1 and probe 2 formed complexes and exhibited fluorescence quenching (Figure 1B). So only a few Cy5 fluorescent spots were observed in the imaging which was caused by incomplete binding of probe 1 and probe 2. When used for detecting miR-126, a large amount of Cy5 fluorescent spots was observed implying that many complexes of probe 1/miR-126 are captured on the surface of the coverslip (Figure 1C). Figure 1D indicated very little false positive signal evoked by non-specific adsorption was observed. These data supported the feasibility of the proposed miR-126 single-molecule imaging method in detecting miR-126. Furthermore, one general fact in the single molecule fluorescence imaging is that all kinds of organic dye molecules including the Cy5 used in our experiments would be photobleached in a few or tens of seconds stochastically. If two or more fluorescent molecules existed in one bright spot, these fluorescent molecules would be photobleached successively rather than simultaneously due the stochastic nature of photobleaching. Consequently, the fluorescence intensity trace of this spot would drop to a lower level and then keep fluctuating whenever one fluorescent molecule is photobleached. Finally, the intensity trace drops to the baseline when all dye molecules are photobleached that generates a multiple-level trace while a single molecule spot would generate a ONE-level trace. All the selected fluorescent spots in our measurements were checked to make sure their intensity traces were one level as shown in Supplementary Figure S1 that guaranteed all the spots were generated by single molecules.

### The sensitivity of this method was optimized by adjusting the concentration and reaction time

The distribution density of probe 1 on the surface of the slide is an important factor in determining the sensitivity of miR-126 examination. Although the high distribution intensity is conducive to the capture of target molecules and increases the detection sensitivity, too-dense distribution affects the discrimination of fluorescent spots in the image, leading to incorrect counting of individual molecules. The results of our optimization of the capture probe concentration showed that 100 pM. was the relatively optimal concentration for producing uniform probe 1 distribution with appropriate intensity. The 10 nM produced an overly dense distribution of capture probes while 1 p.m. did not introduce enough capture probes (Supplementary Figure S2A).

The appropriate concentration of probe 2 is equally important for miR-126 detection, as sufficient probe 2 is required for desaturation to form probe 1/probe 2 complexes. The optimal concentration of probe 2 was 10 nM suggested by the optimization experiment (Supplementary Figure S2B). In addition, we studied the reaction time of miR-126 in order to obtain as many probe 1/miR-126 complexes as possible. As shown in Supplementary Figure S2C, the number of image spots of probe 1/miR-126 complex increased greatly when the binding time was extended to 60 min, while almost reaching saturation after 60 min. Therefore, to balance the efficiency of complex formation and preparation time, we set the reaction time at 60 min for all experiments.

### The detection limit of miR-126 samples in this method

**FIGURE 2:**
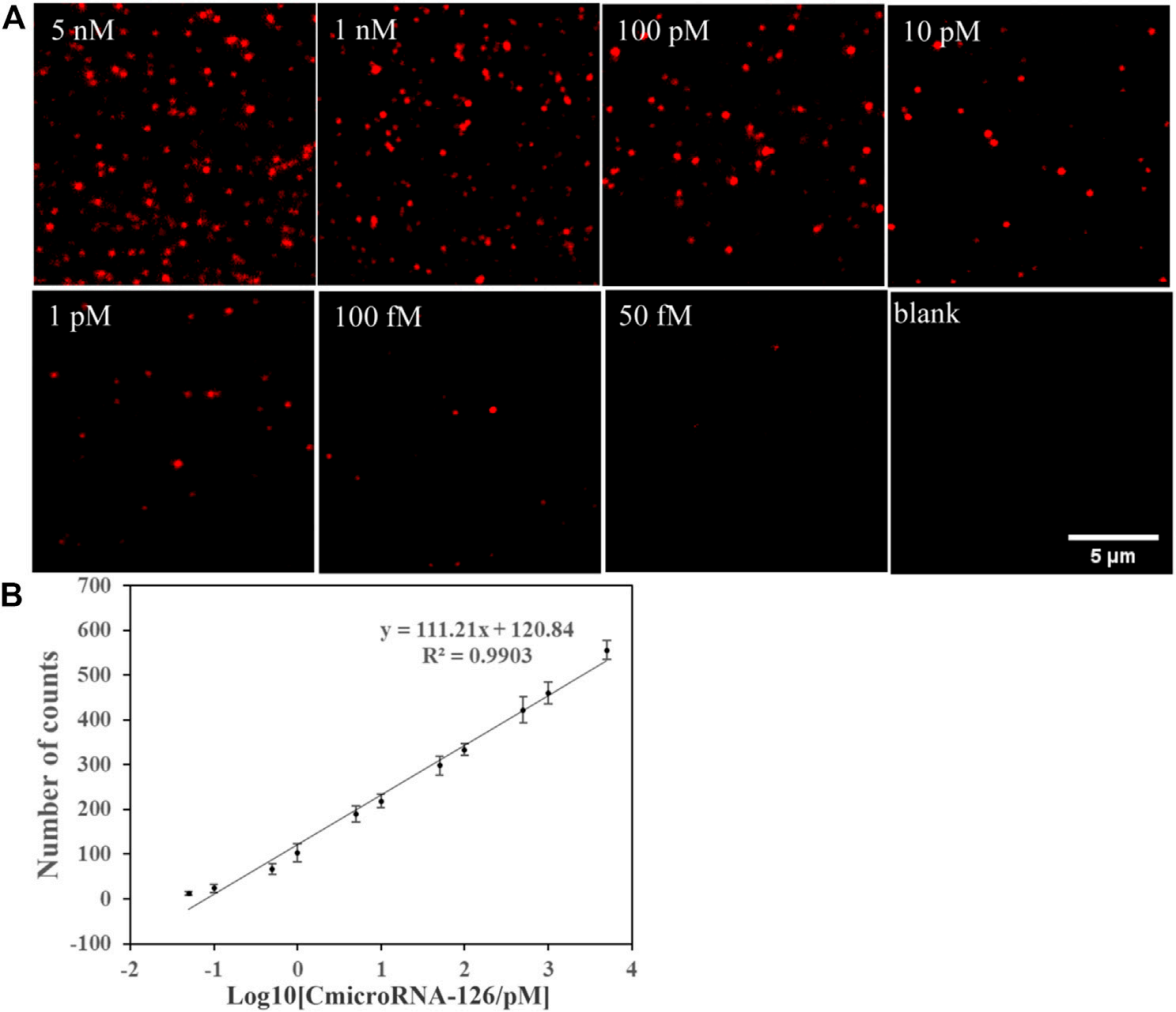
**(A)** Single-molecule fluorescence imaging method was used to detect miR-126 in a series of concentrations of miR-126 samples to find the detection limit. **(A)** Representative Cy5 spots image. **(B)** Correlation between Cy5 spot counts and the logarithm of miR-126 sample concentrations; Scale bar: 5 μm; Error bars indicate the standard deviation of three experiments.

In the optimized condition, the single-molecule imaging method was used to detect miR-126 in miR-126 samples at a range of concentrations. Ten different regions of each sample were selected to quantify the number of miR-126 to reduce the sampling error. From the fluorescent image, we observed that the Cy5 spot number was grown with the rising of miR-126 concentration and the detection limit was 50 fM (Figure 2A). The results of statistical analysis showed that the logarithm of the miR-126 concentration exhibited a linear relation with Cy5 spot counts (Figure 2B). The linear regression equation was N = 111.21Log10 [CmicroRNA-126/pM] + 120.84 (R2 = 0.9903) (Figure 2B). These data suggest that this method does not require signal amplification for the detection of miR-126.

### The selectivity of this method for miR-126 detection

To assess the selectivity of the method, we compared the detected Cy5 spot counts between the miR-126 sample and base mismatched miR-126 (single base or three bases) or non-complementary miRNAs (miR-221, miR-16, miR-143 and miR-141). As shown in Supplementary Figure S3 and Figure 4, the Cy5 image spot counts of the miR-126 sample were 2.1, 8.8, and 26.9–41.3 times higher than other miRNA samples, respectively.

### The discrepancy of MiR-126 abundances of breast cancer cell and control cell

**FIGURE 3:**
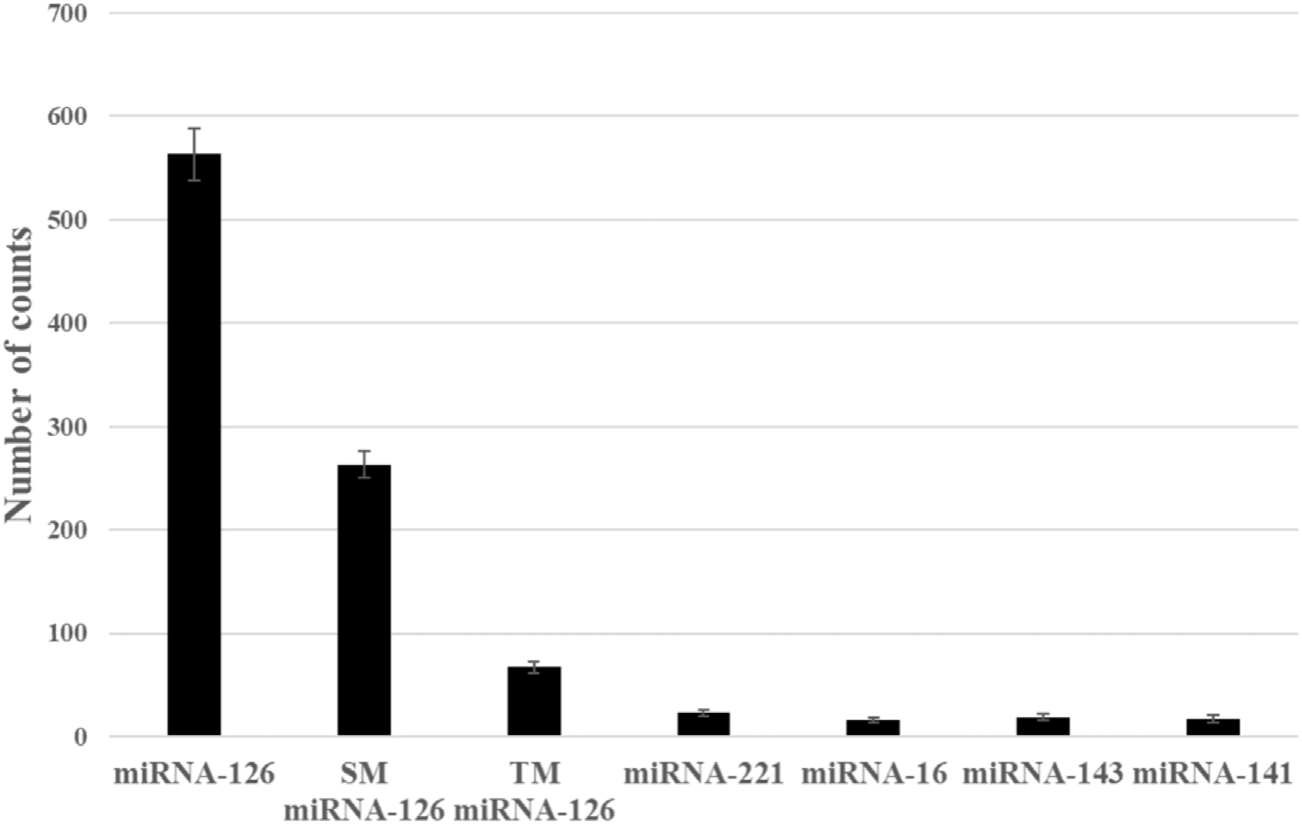
Detection specificity was evaluated by comparing Cy5 counts between the miR-126 sample and base mismatched miR-126 (single or three bases) sample or non-complementary miRNAs (miR-221, miR-16, miR-143 and miR-141) sample. Error bars indicate the standard deviation of three tests.

As in previous studies, miR-126 was expressed in human breast cancer cell MB-231 and normal cell MCF10A (Volinia et al., 2006; Ma et al., 2007; Tavazoie et al., 2008; Png et al., 2012). To assess the detection capability, the method was applied to examine miR-126 levels in MB-231 and MCF10A cells. Figure 3 showed that Cy5 counts showed a linear relation with the logarithm of the cell number. In MB-231 cells, miR-126 counts were fitted by a linear equation N = 58.046 log10 [cell number]—120.14 (R2 = 0.9857) (Figure 3) and that in MCF10A cells was by equation N = 59.976 log10 [cell number] - 45.627 (R2 = 0.9953) in the range of 100–100,000 cells (Figure 3). The detection limit of miR-126 was 100 cells in both cell lines. miR-126 level in the MCF10A cells was higher than that in the MB-231 cells as indicated by the upper position of the fitting curve in the same range, which was consistent with previous reports (Volinia et al., 2006; Ma et al., 2007; Tavazoie et al., 2008; Png et al., 2012). These data suggested that the method has the capability to effectively detect miRNA of cancer cells though cell extraction and determine the discrepancy of miRNA expression level between cancer and control cells.

## Discussion

**FIGURE 4:**
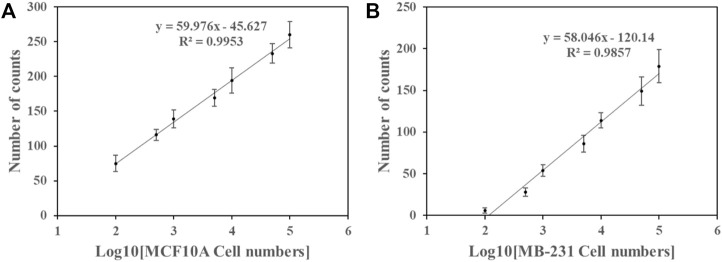
The number of Cy5 spot counts and the logarithm of the cell number counts exhibited a linear relation in the samples of **(A)** MCF10A cells and **(B)** MB-231 cells. Error bars represent the standard deviation of three tests.

In this study, an ultrasensitive single-molecule fluorescence imaging based method to detect miRNAs with capturing probes was proposed (Kim et al., 2012; Yuan et al., 2016; Yu et al., 2017). The optimized condition was determined by screening the optimal reaction concentration and reaction time of the two probes. The detection limit of miR-126 under this optimal condition was determined to be in the range of fM while the specificity was also evaluated. The good signal repeatability of this method was indicated by the standard deviations of all the measurements of varying types of samples in the plots of Figure 2B, Figure 4 and Figures 4A, 4B. The storage stability of samples in our setup can last at least 1 h that is far long enough to finish the measurement. We further validated this proposed method in detecting miR-126 of the extraction of breast cancer and control cells and detected a higher miR-126 abundance in the MCF10A cell than that in the MB-231 cell, which was consistent with previous reports (Volinia et al., 2006; Ma et al., 2007; Tavazoie et al., 2008; Png et al., 2012). Our method reached a significantly higher sensitivity than most traditional non-single molecule methods to detect miRNAs. It also has some advantages over other single molecule detecting methods of mRNAs developed by us and other groups. Firstly, the current method reduced the cost and improved the capturing efficiency of miRNAs by using only two DNA probes rather than the antibody of our previous method (Zhang et al., 2020) and the nano structure probes of other methods (Daneshpour et al., 2016; Li et al., 2019). Secondly, the design of quenching the fluorescence of miRNA unbound probes and its recovering with the capturing of target miRNAs lowered the background noise and the probability of false positive events. However, our method faced big challenge in the *in situ* imaging of miRNAs in live cells because the fluorescence of Cy5 was not very bright. We need to suppress the background noise as well as enhance the fluorescence of probes in our future research.

In conclusion, our single-molecule miRNA imaging method showed high specificity and sensitivity for miR-126. By designing different probe sequences to capture different miRNAs, this method can be developed into an ultra-sensitive universal miRNA detection method that has broad prospects of application in tumor diagnosis.

## Data Availability

The original contributions presented in the study are included in the article/Supplementary Material, further inquiries can be directed to the corresponding authors.
