# Supplementary material for: Evaluation of probe-based ultra-sensitive detection of miRNA using a single-molecule fluorescence imaging method: miR-126 used as the model
Source: Front Bioeng Biotechnol. 2023 Jan 24;11:1081488. doi: 10.3389/fbioe.2023.1081488 (PMC9902880; doi:10.3389/fbioe.2023.1081488)
Supplement: Supplementary file 1 [file Table1.docx]

**Supplementary Table S1.** Nucleotide sequences of miRNAs and probes used in this assay.

| Name | Sequence (5’-3’) |
| --- | --- |
| miR-126 | UCG UAC CGU GAG UAA UAA UGC G |
| Single-base mismatch miR-126 (SM miR-126) | UCG UAU CGU GAG UAA UAA UGC G |
| Three-base mismatch miR-126 (TM miR-126) | UCG UAU CGU UAG UAA UAA UUC G |
| miRNA-221 | AGC UAC AUU GUC UGC UGG GUU UC |
| miRNA-16 | UAG CAG CAC GUA AAU AUU GGC G |
| miRNA-143 | UGA GAU GAA GCA CUG UAG CUC A |
| miRNA-141 | UAA CAC UGU CUG GUA AAG AUG G |
| Probe 1 | Biotin-C GCA TTA TTA CTC ACG GTA CGA TGT CGC TT-Cy5 |
| Probe 2 | BHQ2-GCG ACA TCG TA |
